# Differential power of placebo across major psychiatric disorders: a preliminary meta-analysis and machine learning study

**DOI:** 10.1038/s41598-021-99534-z

**Published:** 2021-10-29

**Authors:** Bo Cao, Yang S. Liu, Alessandro Selvitella, Diego Librenza-Garcia, Ives Cavalcante Passos, Jeffrey Sawalha, Pedro Ballester, Jianshan Chen, Shimiao Dong, Fei Wang, Flavio Kapczinski, Serdar M. Dursun, Xin-Min Li, Russell Greiner, Andrew Greenshaw

**Affiliations:** 1 Department of Psychiatry, Faculty of Medicine and Dentistry, University of Alberta, Edmonton, AB Canada; 2 Department of Computing Science, Faculty of Science, University of Alberta, Edmonton, AB Canada; 3 Department of Mathematical Sciences, Purdue University Fort Wayne, Fort Wayne, US eScience Institute, University of Washington, Seattle, USA; 4 Department of Psychiatry and Behavioural Neurosciences, McMaster University, Hamilton, ON Canada; 5 Laboratory of Molecular Psychiatry, Hospital de Clínicas de Porto Alegre, Programa de Pós-Graduação em Psiquiatria e Ciências do Comportamento, Universidade Federal do Rio Grande do Sul, Porto Alegre, Brazil; 6 Neuroscience Graduate Program, McMaster University, Hamilton, ON Canada; 7 Early Intervention Unit, Department of Psychiatry, Affiliated Brain Hospital of Nanjing Medical University, Nanjing, Jiangsu China; 8 Amii (Alberta Machine Learning Institute), Edmonton, AB Canada

**Keywords:** Machine learning, Bipolar disorder, Depression, Schizophrenia

## Abstract

The placebo effect across psychiatric disorders is still not well understood. In the present study, we conducted meta-analyses including meta-regression, and machine learning analyses to investigate whether the power of placebo effect depends on the types of psychiatric disorders. We included 108 clinical trials (32,035 participants) investigating pharmacological intervention effects on major depressive disorder (MDD), bipolar disorder (BD) and schizophrenia (SCZ). We developed measures based on clinical rating scales and Clinical Global Impression scores to compare placebo effects across these disorders. We performed meta-analysis including meta-regression using sample-size weighted bootstrapping techniques, and machine learning analysis to identify the disorder type included in a trial based on the placebo response. Consistently through multiple measures and analyses, we found differential placebo effects across the three disorders, and found lower placebo effect in SCZ compared to mood disorders. The differential placebo effects could also distinguish the condition involved in each trial between SCZ and mood disorders with machine learning. Our study indicates differential placebo effect across MDD, BD, and SCZ, which is important for future neurobiological studies of placebo effects across psychiatric disorders and may lead to potential therapeutic applications of placebo on disorders more responsive to placebo compared to other conditions.

## Introduction

Placebo is a sham medicine or procedure without active chemical or physical ingredients^1^. In clinical trials, placebos are generally control treatments similar to the studied intervention but without their active ingredient. However, placebo may affect clinical outcomes through psychosocial interactions, which can lead to a high degree of therapeutic effectiveness^2^. Although it remains unclear whether the placebo effect is equally powerful for all diseases^3,4^, the effect is often large in psychiatric disorders. For example, the placebo effect in the major depressive disorder (MDD) could be comparable to the pharmaceutical effect from antidepressants, sometimes as large as over 80%^5–7^. Common patterns of glucose metabolism changes in cortical and paralimbic regions metabolism were identified in unipolar depressive patients responding to placebo and an antidepressant^8^. Various neurobiological mechanisms of placebo effect have been revealed in neurological and psychiatric conditions^9–11^, but for psychiatric disorders, most of the studies focused on depression^12^. Other factors contributing to the placebo effect in psychiatric disorders were revisited based on findings from individual conditions, and low baseline symptom severity, more recent trials, and unbalanced randomization were associated with high placebo effect^13^.

Understanding the placebo effects across psychiatric disorders may help us understand the pathological and therapeutic mechanisms underlying these disorders and their corresponding treatments, and provide insights that may guide the use of placebo as a control condition in clinical research or as an active component in mental health practice targeting different conditions^7^. However, few studies, if any, have directly compared the placebo effect across multiple psychiatric disorders, while considering the confounding effects of different interventions and different assessments of symptoms. If placebo effects are indeed reliably different in psychiatric disorders, would it be possible to categorize these disorders based on their corresponding placebo effects using machine learning? This is another way to demonstrate whether differential placebo effects exist across psychiatric disorders.

Here, we investigated whether placebo effects were reliably different across the major psychiatric disorders, including schizophrenia (SCZ), bipolar disorder (BD) and MDD. We conducted preliminary meta-analyses and machine learning analyses of different measures of the placebo effect based on existing clinical trials data from clinicaltrials.gov. Our hypotheses are (a) patients with MDD, BD depression, BD mania and SCZ have differential placebo effects, and (b) trials involving these major psychiatric disorders are distinguishable from one another based on their respective placebo effects.

## Results

### Results of screening

**Figure 1:**
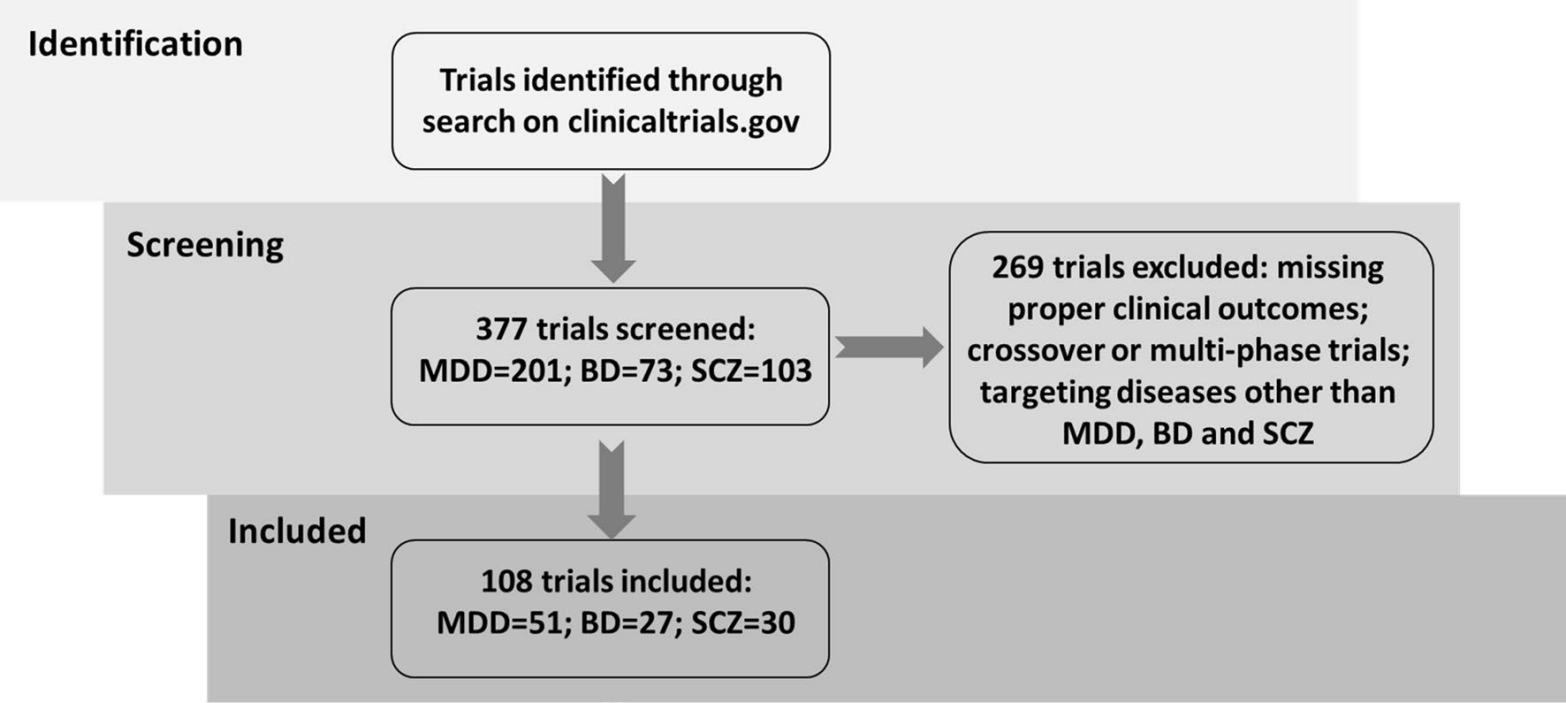
Searching and screening process of clinical trials. *MDD* major depressive disorder, *BD* bipolar disorder, *SCZ* schizophrenia.

We identified 201 trials for MDD, 73 for BD, and 103 for SCZ after screening of the search results. After applying our exclusion criteria, 51 MDD trials, 27 BD trials, and 30 SCZ trials were included in the analyses (Fig. 1). These trials were conducted between 1996 and 2016 and involved a total of 32,035 participants (n intervention = 17,435; n placebo = 14,600). The descriptive characteristics are summarized in Table 1 (for all the detailed information of the trials, please refer to the electronic supplementary material).

**Table 1:** Descriptive table of clinical trials included in the meta-analysis.

	MDD (N = 51; total sample 17,621)				SCZ (n = 30; total sample 7869)				BD (n = 27; total sample 6545)			
	Mean	Std	Min	Max	Mean	Std	Min	Max	Mean	Std	Min	Max
N placebo	153.23	81.67	5	300	119.21	51.59	19	208	112.26	73.72	7	230
Randomization (intervention/placebo)	1.08	0.47	0.32	4.20	1.00	0.30	0.34	2.27	1.03	0.32	0.20	2.01
Age intervention mean	40.94	12.96	12.60	72.89	39.29	7.99	15.4	50.1	38.50	9.48	11.7	49.3
Age placebo mean	41.30	12.55	12.60	73.02	38.70	8.53	15.4	48	38.56	9.39	11.8	46.6
Sex ratio intervention (M/F)	0.78	0.66	0	4.50	2.25	1.48	0.70	8	0.95	0.38	0.40	2.00
Sex ratio placebo (M/F)	0.70	0.35	0	2.29	1.99	0.82	0.82	4.21	0.92	0.39	0.40	2.00
Number of facilities	42.25	25.92	1	100	50.27	30.26	1	115	33.75	37.73	1	130
Time duration (weeks)	8.51	1.58	6	13	7.46	2.68	6	16	7.85	2.17	6	12
Start year	2009	2	2005	2013	2009	4	1996	2014	2008	2	2004	2012
Completion year	2011	2	2007	2016	2011	3	2002	2014	2011	2	2008	2016
Number of visits	6.03	0.83	2	14	6.41	2.94	2	13	7.11	3.35	2	21

### Measures of placebo effect across disorders

Clinical trials assessing different disorders used distinct clinical assessments, which are not directly comparable due to different score ranges. This is especially challenging when baseline severity and typical treatment responses across disorders are different. The symptom improvement from placebo or intervention could be calculated as the decrease of the corresponding clinical assessment after placebo or intervention compared to the baseline. However, the decrease of a clinical scale for SCZ may not be comparable numerically to the decrease of a clinical scale for MDD (Fig. S1). The standardized mean difference (SMD) is commonly used, which calculates the effect size of the intervention or placebo in each study relative to the variability observed in that study. However, the SMD assumes the differences in standard deviations to be purely from differences in the outcome measurements among studies but not from the variability among the study populations^14^. This assumption may hold to some extent when SMD is applied to data from clinical trials of the same disorder, but it may not be valid for cross-disorder comparisons when variability of the outcome measurements may include variance from both measurement themselves and the different patient populations. To account for these challenges, we developed measures using the decrease in clinical assessment after active treatment as well as the baseline assessments as references and considered the ratio of the decrease in clinical assessment due to placebo to these references to compare the placebo effect across disorders. Clinical scales that are based on a mixture of self-reported symptoms, objective measures and clinician evaluations may have different characteristics compared to those based on clinician’s subjective impression (e.g., CGI-S), so we also used relative CGI-S change in addition to the clinical assessments, which is comparable across the conditions. Thus, in our study, we developed three different outcome measures for placebo effects, including measures involving patient reported symptoms and clinician evaluation that are typically not comparable, and measures that were based on subjective clinician assessment that was comparable across disorders (e.g., CGI-S). Two measures of the placebo effect were scaled to the corresponding intervention effect (Fig. S2), and one was compared to the baseline. The ratios for trials that included two active interventions were calculated separately. We used the following ratios to evaluate the placebo effect across psychiatric disorders:

- $R_{clinical} = \frac{{\Delta Clinical\;Scales_{Placebo} }}{{\Delta Clinical\;Scales_{Active\;Drug} }}$, the ratio of the average clinical measurement change from baseline for placebo to the active drug; the $\Delta Clinical\;Scales$ was calculated as the baseline measurement minus the endpoint measurement to indicate a decrease of the symptoms.
- $R_{CGI} = \frac{{\Delta CGI_{Placebo} }}{{\Delta CGI_{Active\;Drug} }}$, the ratio of the average CGI-S change from baseline for placebo to the active drug; the $\Delta CGI$ was calculated as the baseline CGI-S minus the endpoint CGI-S to indicate a decrease of the clinical severity.
- $R_{CGI\;Basline} = \frac{{\Delta CGI_{Placebo} }}{{CGI\;Basline_{Placebo} }}$, the ratio of the average CGI-S decrease at the end of the study to the average CGI-S baseline for placebo.

### Results of meta-analysis

**Figure 2:**
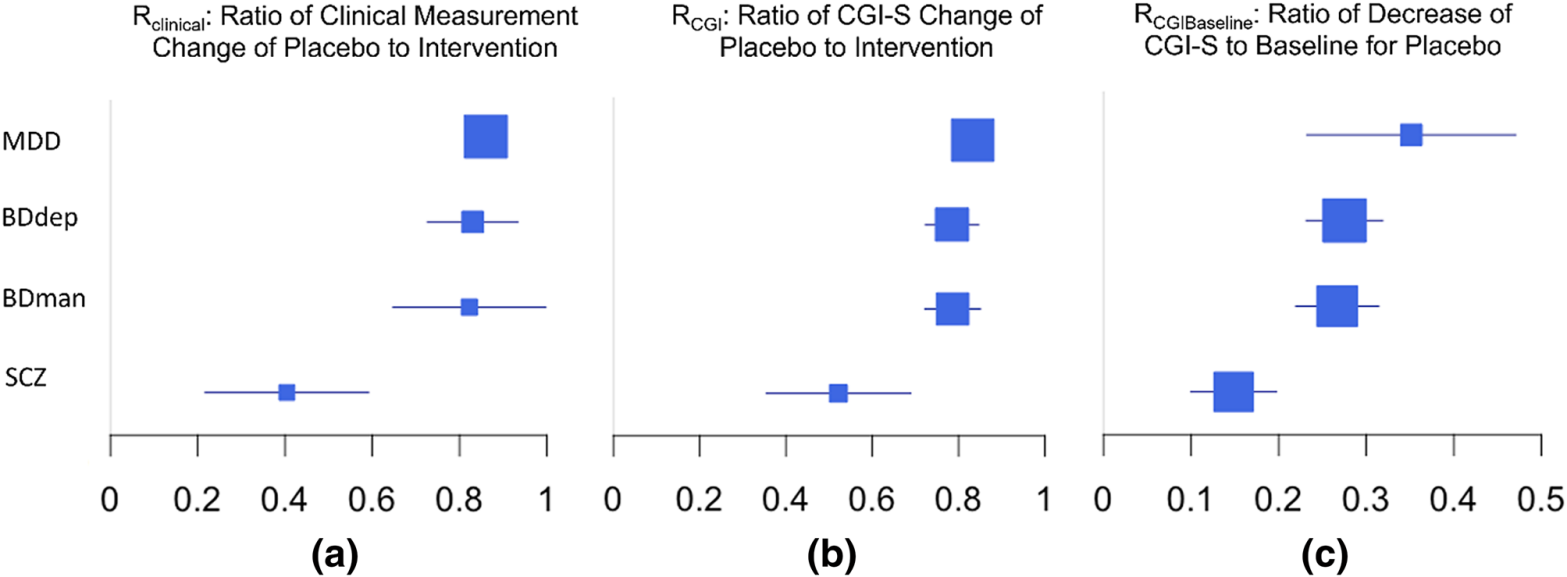
Differential placebo effect for MDD, bipolar disorder-depression (BDdep), bipolar disorder-mania (BDman), and SCZ, as measured by $R_{clinical}$, $R_{CGI}$ and $R_{CGI\;Basline}$. The box size indicates power estimates, a larger box representing a smaller range of confidence interval.

By performing meta-analysis on the original data, we found that SCZ showed a lower placebo effect when compared to mood disorders, as shown in Fig. 2 (*P* values for all three ratios when SCZ was compared to the other conditions < 0.001, all Hedge's g > 0.685^15^).

**Figure 3:**
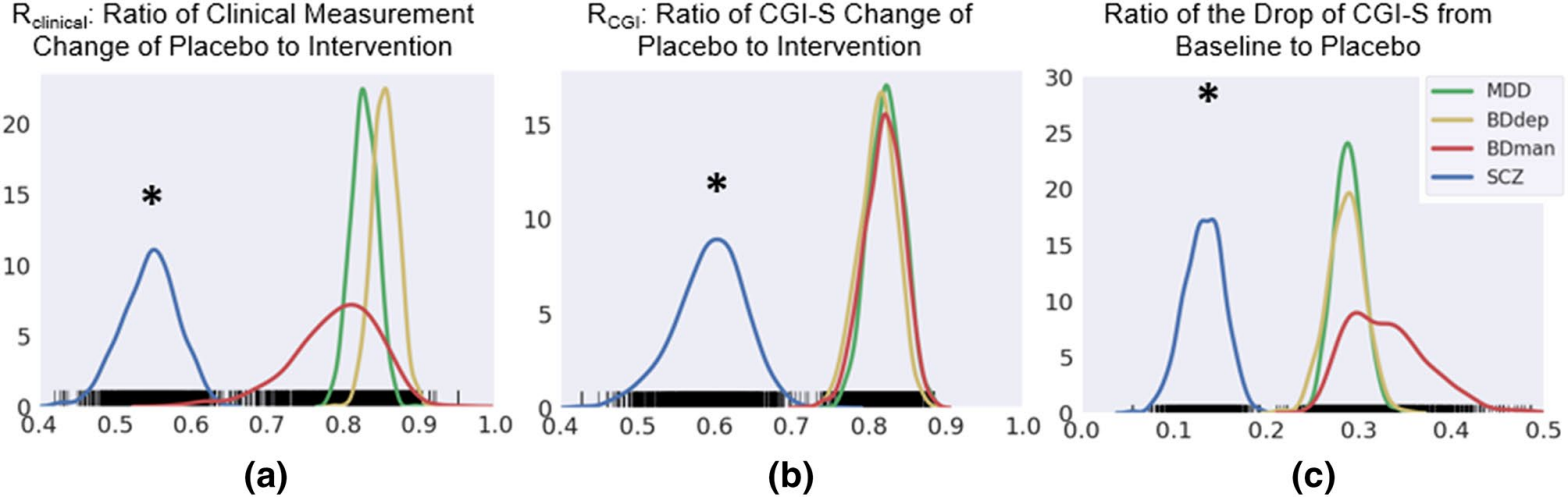
Differential placebo effect for SCZ, BDman, BDdep and MDD, as confirmed by sample-size weighted bootstrapping using (**a**) the ratio of clinical measurement change from baseline for placebo to intervention, (**b**) ratio of CGI-S change from baseline for placebo to intervention, and (**c**) ratio of CGI-S decrease from baseline to the CGI-S value at the baseline for placebo. The placebo effect was always greater than zero, while less than one, meaning patients could not fully recover or achieve improvement comparable to intervention by just taking placebo. The placebo effect for SCZ is significantly lower than that for MDD and BD. *Denotes significant difference from other distributions at *P* < 0.001.

By performing meta-analysis with weighted bootstrap resampling (WBR), we found that SCZ showed a smaller placebo effect compared to mood disorders (e.g., MDD, BDdep and BDman), as shown in Fig. 3 (for each of the three paired comparisons, *P* < 0.001, Hedge’s g > 0.80; Table S1). We also found consistent placebo effects on all four psychiatric conditions across the three measures (one sample t tests against zero, *P* < 0.001, Hedge’s g > 0.79), and significantly less efficacy of placebo when compared to the active drug (one sample t tests against one for $R_{clinical}$ and $R_{CGI}$, *P* < 0.001, Hedge’s g > 1, with the exception of BDman, with a hedge’s g of 0.33).

### Results of meta-regression

The only variable with a consistently strong negative coefficient was SCZ versus MDD (*P* < 0.001), indicating an association with lower placebo effect in SCZ compared to MDD. The other variables significantly different from zero were: BD depression (*P* < 0.001), mania (*P* < 0.001), phase (*P* = 0.013), being used as co-treatment (*P* < 0.001), number of facilities (*P* = 0.004) and number of study arms (*P* < 0.002) (all measured by ${R}_{CGIBasline}$, BD depression, mania, later phase and higher number of facilities associated with lower placebo effect, while being used as co-treatment and higher number of visits associated with higher placebo effect); being conducted in North America (*P* = 0.002) was associated with higher placebo effect as measured by $R_{clinical}$. These results are summarized in Tables S3–S5 in supplementary materials.

The main result of differential placebo effects between SCZ and MDD was confirmed with WBR meta-regression across all three placebo effect measures (*P* < 0.01; Table S2). No other factor was consistently associated with placebo effect across the three measurements.

These results confirmed that the meta-analysis findings were not due to other potential confounding factors, and that SCZ was associated with lower placebo effect when compared to mood disorders. It is worth to note that according to the current regression results, the placebo effect was not associated with (1) whether the trial was conducted by academic institutions or industrial companies, (2) whether the trial was in Phase 3 or 4, or (3) whether the trial recruited patients with the typical psychiatric disorder or special samples, such as patients with residual symptoms or that are treatment resistant.

### Results of classification using placebo effect

In addition to the statistically significant difference of placebo effect in MDD, BD and SCZ, we were interested in whether the placebo effect provided further “predictivity” of the condition that each trial was associated with, because significant difference does not automatically lead to good prediction or distinguishment of individual cases^16^ but a good distinguishment of individual cases can demonstrate reliable differentiation between the conditions with respect to placebo effects. This could be assessed with a classification task using machine learning based on the three measures of the placebo effect between the three conditions. The individual-trial level classification based on placebo effect may lead to further applications of placebo effect in recognizing phenotypes with respect to their responsiveness to placebos.

### Original data

**Figure 4:**
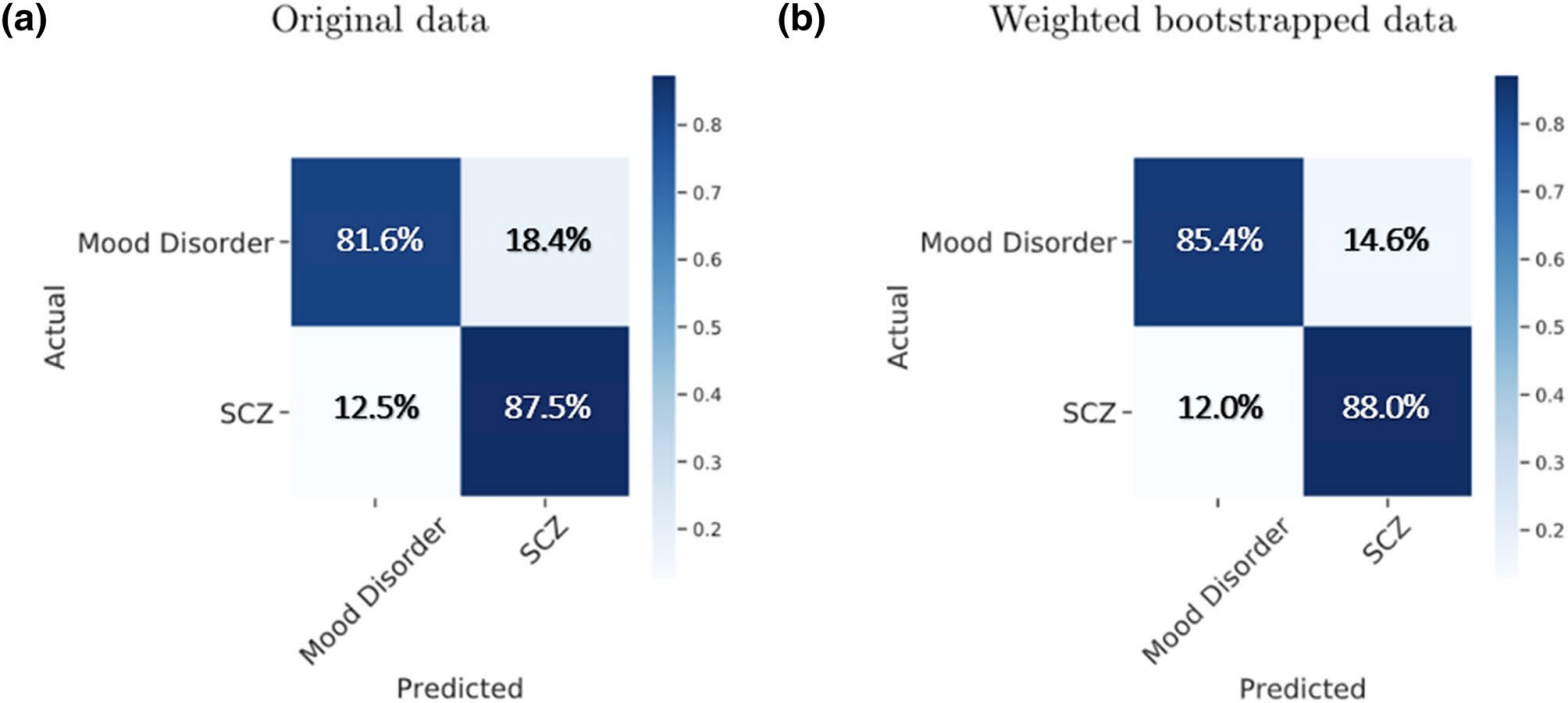
Confusion matrix of L1 penalized logistic regression classification model based on (**a**) the original data, and (**b**) the weighted bootstrapped data. Both results confirmed that we could identify SCZ and mood disorders at the individual-trial level based on the three measures of placebo effect. Mood Disorder consists of MDD, BDdep and BDman, while SCZ denotes Schizophrenia.

We retained the trials with all three ratios as valid for follow-up classification analysis (73 trials). We obtained an average balanced accuracy (the average of sensitivity and specificity) of 84.6% when classifying SCZ and mood disorders (*χ*^2^ (3) = 9.19, *P* < 0.05; sensitivity for SCZ, 87.5% and specificity 81.6%; Fig. 4). A three-way classification of MDD, BD and SCZ could distinguish SCZ from MDD and BD, but could not distinguish MDD from BD (Fig. S3).

### WBR data

On each of the 1000 WBR datasets, we retained trials with all three ratios valid for classification analysis (78,374 trials). We achieved an average balanced accuracy of 86.7% (*χ*^2^ (3) = 12,920, *P* < 0.001) to distinguish SCZ and mood disorders (sensitivity for SCZ, 88.0% and specificity 85.4%; Fig. 4).

## Discussion

In the current study, we investigated 108 clinical trials comprising 32,035 participants. By using three measures to evaluate the placebo effect and applying several analytical approaches, we found differential placebo effects across three major psychiatric disorders, and the placebo effect was significantly lower in SCZ than mood disorders. The differential placebo effect can also be used to distinguish SCZ from mood disorders trials at the individual trial level using machine learning, which was a more challenging task compared to detecting group-level statistical significance and provides a stronger evidence that the placebo effect must be reliably different in SCZ and mood disorders. To our best knowledge, this is the first study to show converging evidence of differential placebo effects across major psychiatric disorders from different measures and different analytical approaches. Disorder-specific placebo effect may suggest different pathological and therapeutic mechanisms of placebo underlying major psychiatric disorders and corresponding treatments. Our study may provide an approach to estimate the magnitude of the placebo effect in different psychiatric disorders when placebo a control condition in clinical trials, or enable its use as an active component along with other treatments in mental health practice^6^.

**Figure 5:**
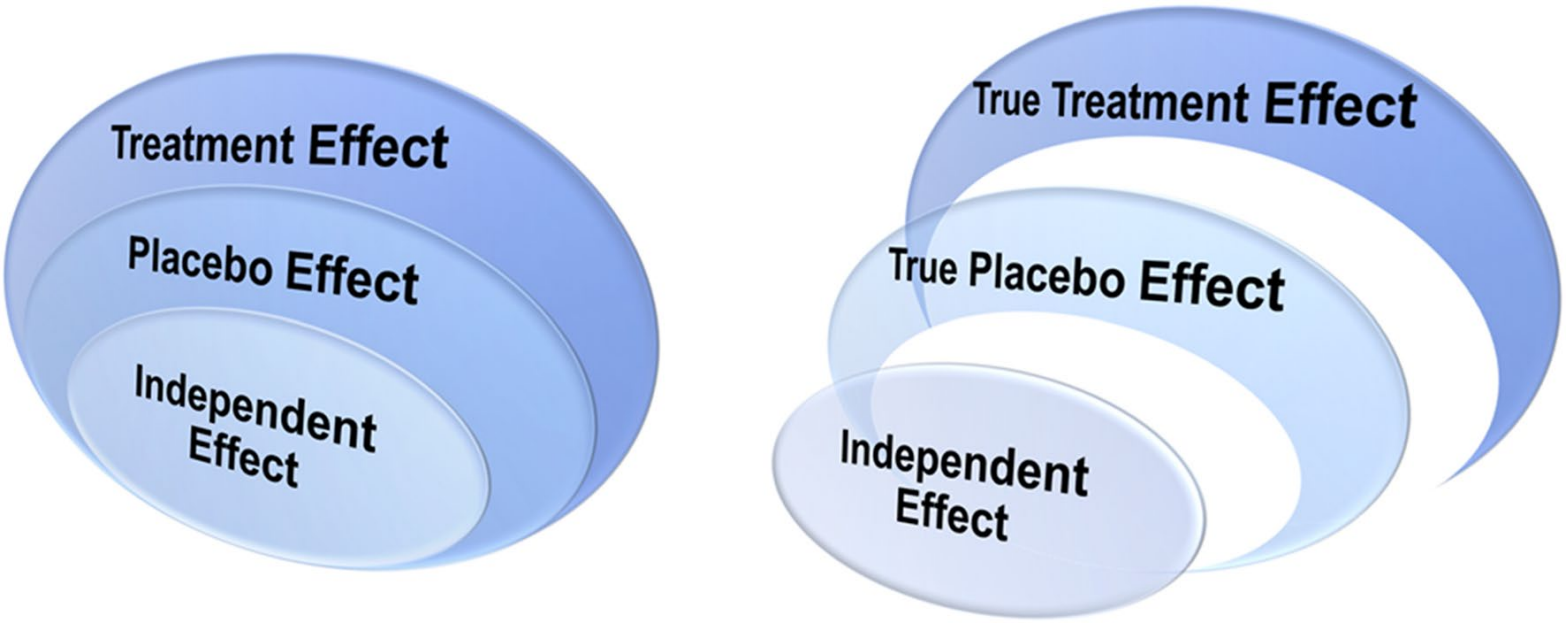
The additive model of placebo effect. The observed treatment effect includes the observed placebo effect, while the observed placebo effect includes independent effect (e.g., spontaneous improvement and natural course of the disease). In an additive model, the true treatment effect is the observed treatment effect subtracting the true placebo effect and the independent effect, and the true placebo effect is the observed placebo effect subtracting the independent effect.

Observed treatment effect is considered to include an observed placebo effect, while that placebo effect, in turn, includes independent effect, e.g., spontaneous improvement and natural course of the disease^4,17^. In an additive model, true placebo effect is considered as the observed placebo effect “minus” the independent effect, while the true treatment effect is the observed treatment effect “minus” the true placebo effect and the independent effect (Fig. 5) ^17^. In fact, the treatment effect, placebo effect, and independent effect may all scale differently according to the disorder and measurement types. Thus, in our study, we used two ratios of clinical assessments, both relative to the observed treatment effect, instead of just using the changes of these scales in the placebo group alone. CGI-S changes relative to baseline were not scaled to treatment measurements but directly comparable across disorders, and thus were complementary to those two ratios relative to the treatment. All three ratios consistently showed differential placebo effect across the major psychiatric disorders, especially between SCZ and mood disorders.

The present study focused on the observed placebo effect across major psychiatric disorders, instead of the true placebo effect, as seen in several previous studies^4,18^. Investigating the observed placebo effect is valuable since it contributes significantly to the overall treatment effect. Our study showed that the overall placebo effect can be as large as 70–90% of the treatment effect in mood disorders, while only around 50–60% in SCZ according to the ratios between placebo/active treatment (Fig. 3). This indicates that the placebo may have a greater effect on mood disorders than on SCZ. Thus, improving factors contributing to the placebo effect, such as skillful consulting and attention to the doctor-patient relationship, may be a cost-effective way to advance the care of mood disorders^19^.

Previous studies have identified several predictors of larger placebo effects, such as lower baseline symptom severity, more recent trials, and unbalanced randomisation (more patients randomly assigned to drug than placebo)^13,20^, which may be associated with higher expectations^19,21,22^. Some of these predictors may have distinct effects on different disorders. For example, mixed results have been observed regarding baseline symptom severity. Some studies found that lower baseline severity of psychotic symptoms might be associated with a higher placebo effect, while other studies found the opposite^13,23^. Because most previous studies evaluated the placebo effect for a specific disorder and then compared the post hoc effect of predictors independent of disorder types, it was difficult to assess the contribution of these predictors across different disorders. In the present study, our meta-regression models included most of the common predictors along with disorder types, and we did not find consistent predictors across placebo effect measures other than diagnoses. Thus, future studies with large samples and including all potential predictors comparable across psychiatric disorders, will be necessary to identify reliable predictors of placebo effect and to investigate the potential interaction between the disorder types and other predictors.

One potential factor that may contribute to the differential placebo effect in SCZ and mood disorders is the patient's insight and awareness of the disease, as studies have shown that active placebos may have stronger effect than inert placebos^24^. Impaired insight is one of the hallmark features of SCZ, and may be implicated in the lower placebo effect we found^25^. In a 1-year observational study of patients with SCZ and BD, higher insight was associated with higher medication adherence scores and stronger therapeutic alliance^26^. In addition, a cross-sectional study of SCZ and BD patients showed that around 40% of SCZ patients were unaware of their disorder, while no patient in the BD group was unaware of their disorder^27^. The differential insight and awareness found in these disorders may affect the expectation of treatment response and other psychological processes, which is in accord with a previous observation on psychotic depressive patients, who were less responsive to placebos compared to those without psychosis^28^.

The current study has limitations. Compared to other typical meta-analyses based on scientific literature, our preliminary study is an analysis of the existing analyses from clinical trials reported on the publicly available clinicaltrials.gov registry, a meta-analysis in a broad sense. A future thorough study that considers hybrid levels of resources including registered clinical trials, publications and private datasets will be necessary to validate the study findings. We did not focus on trials with a no-treatment group, which was considered as a reference condition to exclude the independent effects from the observed placebo effect. The no-treatment condition is difficult to implement in clinical trials of psychiatric disorders due to enrollment and ethical issues. The number of trials with no-treatment group was limited for clinical trials of psychiatric disorders, and existing conclusions about “true placebo effect” in psychiatry is based on such trials. Furthermore, the additive model of the placebo effect still needs validation, and the effect of no-treatment may involve interactions between patients and service providers other than the official treatment procedure or contributions from factors like the Hawthorne effect, where the condition of simply being observed will change behaviour or expectations. The lack of differentiation between BD and MDD could be due to smaller sample sizes of BD trials, as well as the overlap of clinical assessments and impressions for these two disorders. Our results were derived from clinical trials that depended on the common interaction between clinicians and patients with the expectation to improve the symptoms and did not take into consideration of scenarios when placebo could turn to “nocebo”, where the expectations were negatively associated with symptom improvement. The limitations of clinical trials will affect our results (See Supplementary Materials). We also could not differentiate what was defined by previous studies as “placebo response” and “placebo effect”^19,21^. However, the placebo effect in our study was represented with three distinct measurements including comparison to active treatment and to the baseline status, which provides a multi-perspective view of the placebo effect and confirms the consistency of our results. While our study was among the first to investigate placebo effect in mental illnesses, it did not consider all mental disorders—we anticipate future studies will explore other diseases, including anxiety, obsessive–compulsive, substance use disorders and other mental disorders and comorbidities.

In the current study, we found converging evidence that the power of the placebo effect is different across psychiatric disorders. By using various measures of differential placebo effects, we were also able to distinguish SCZ trials from mood disorders trials. These findings suggest potentially distinct mechanisms of placebo underlying MDD, BD and SCZ. The differential placebo effect can guide how placebo can be used as a control condition in clinical trials for these disorders. It can also provide insights of placebo use as a cost-effective active component in future practice in mental health. Our results call for future studies on common and distinct neurological markers of placebo effect across psychiatric disorders, and translational applications of placebo in the frame of personalized medicine.

## Methods

### Search strategy and selection criteria

We performed a systematic search for clinical trials investigating pharmacological interventions on MDD, BD and SCZ at clinicaltrials.gov in December, 2018. We applied the following filters to the search in addition to the disorder types: “Completed Studies, Studies With Results, Interventional Studies, Placebos, Phase 3, Phase 4”. For MDD, we used the term “depression” to include all potential trials that may be related to MDD. Trials that were found in both bipolar and depression search results but only enrolled patients with bipolar depression were considered as BD trials.

We excluded trials without a clinical outcome suitable for the present analysis (e.g., trials addressing only maintenance and/or relapse or missing outcome measurement scales), crossover or multi-phase trials, and trials addressing patients with other diseases.

### Data extraction

The characteristics extracted from each study were the National Clinical Trial Identifier (NCT ID), phase (Phase 3 or 4) and duration of the trial, specific condition (typical or residual symptoms including treatment-resistant disorders), as co-treatment (intervention and placebo were used as co-treatment), start and completion date, continents (the continental regions where the trial was performed: North America, South America, Europe, Asia, Africa, and/or Oceania), the number of subjects in the intervention and placebo groups that started and finished the study, the numbers of female and male subjects in the intervention and placebo groups, the mean and standard deviation of age in the intervention and placebo groups, the number of facilities, countries, states, agency, agency type (academia or industry), the number of total patient visits, the number of arms of the study, and the clinical scale used to measure the intervention and placebo outcome [e.g., Positive and Negative Syndrome Scale (PANSS)^29^, Hamilton Depression Rating Scale (HDRS)^30^, The Montgomery-Åsberg Depression Rating Scale (MADRS)^31^, Young Mania Rating Scale (YMRS)^32^ or Clinical Global Impression-Severity scale (CGI-S)^33^, and the mean and standard deviation of clinical scales in the intervention and placebo groups. All extracted data used for analysis are provided in Table S6 in electronic supplementary materials.

### Meta-analysis

We compared $R_{clinical}$, $R_{CGI}$ and $R_{CGI\;Basline}$ of MDD, BD depression (BDdep), BD mania (BDman), and SCZ from the extracted clinical assessments. For trials with two active interventions, we treated them as two separate trials when calculating the ratios because the two interventions provided two different references for the placebo effect. For trials with multiple arms with the same medicine but different dosages, only the highest dosage was used to provide a relatively conservative estimation of the placebo effect, as the highest dosage usually led to the strongest treatment effect. Mann–Whitney U tests were used because most distributions of these ratios did not follow a normal distribution and might be heterogeneous across conditions. The standard errors and 95% confidence intervals were computed using bootstrapping techniques for each disorder. Forest plots were produced based on the distribution by disorders of the corresponding ratios in the extracted original data.

### Weighted bootstrap resampling for meta-analysis

As sample size of included clinical trials varied considerably and trials with a small sample could have less representative results compared to large samples, we considered the sample size difference when we integrated the outcomes. Thus, in addition to the meta-analysis and meta-regression based on measures derived from the original data, we performed further analyses using weighted bootstrap resampling (WBR) to estimate the mean of the placebo effect across disorders (see Supplementary Materials)^34^.

### Meta-regression

To confirm our results and investigate other potential predictors of placebo effect, for each outcome ratio, we performed a meta-regression analysis using the original clinical trial data. The four psychiatric conditions were coded into three binary codes (MDD, BDdep, BDman and SCZ) with MDD as the reference condition. In addition to the four disorders, we included trial characteristic variables other than the clinical scales related to the outcome ratios. Categorical variables were converted to dichotomous, quantitative variables (dummy variables). We considered a *P*-value smaller than 0.05/3 = 0.0167 for the coefficient to be significantly different from zero according to the Bonferroni correction for three comparisons for the three placebo measurements. We also performed a meta-regression based on the WBR method as described above.

### Classification of mood disorders versus schizophrenia using placebo effect

We also aimed to demonstrate that the placebo effect could also enable us to distinguish disorders at the individual-trial level. We applied logistic regression with L1 regularization to the ratios described above ($R_{clinical}$, $R_{CGI}$ and $R_{CGI\;Basline}$) to classify mood disorders (MDD and BD) versus SCZ. Consistent with previous analyses, we also used WBR to estimate the performance of our machine learning model. The hyperparameters and validation procedure are described in Supplementary Materials. We used *χ*^2^ to compare the chance matrix based on observed frequency to our classification confusion matrix, targeting prediction of mood disorders and SCZ separately.

## Supplementary Information

Supplementary Information 1.

Supplementary Information 2.
