# Supplementary material for: Differential power of placebo across major psychiatric disorders: a preliminary meta-analysis and machine learning study
Source: Sci Rep. 2021 Oct 29;11:21301. doi: 10.1038/s41598-021-99534-z (PMC8556377; doi:10.1038/s41598-021-99534-z)
Supplement: Supplementary file 1 — Supplementary Information 1. [file 41598_2021_99534_MOESM1_ESM.docx]

**Supplementary Material for:** Differential Power of Placebo across Major Psychiatric Disorders: A Preliminary Meta-analysis and Machine Learning Study by Bo Cao, et al.

*Corresponding author:

Bo Cao, PhD

Department of Psychiatry

Faculty of Medicine & Dentistry, University of Alberta, Edmonton, AB, Canada

E-mail: cloudbocao@gmail.com

Phone number: +1 (780) 407-6504

**TABLE OF CONTENTS**

**SUPPLEMENTARY TEXT**....................................................................................................2

**SUPPLEMENTARY FIGURES S1-S3**..................................................................................3

**SUPPLEMENTARY TABLES S1–S6**....................................................................................5

**SUPPLEMENTARY TEXT**

**Weighted bootstrap resampling for meta-analysis**

Bootstrapping is a technique that uses re-sampling with replacement from the sample population to perform inference about the true population. To incorporate the sample size difference across the trials, for each disorder, we performed bootstrapping resampling 1000 times. The chance of a trial to be selected was proportional to (weighted by) its sample size in relation to the total number of subjects undergoing clinical trials for the corresponding disorder.

Clinical trials missing the change from baseline of the clinical assessment for intervention or placebo were excluded from the weighted bootstrap resampling (WBR). Clinical trials with an R_clinical_ greater than three standard deviations from the overall mean for a specific disorder were also excluded as outliers.

We generated the distribution of the mean values of the WBR sample for each ratio, and then used the t-test for WBR distributions of means of ratios.

For WBR meta-regression, we used the distribution of t values of each variable over the 1000 bootstrapping samples generated by the WBR to estimate the two-tailed p-value for the corresponding variable. This is a numerical estimation of the significance of contribution of a specific variable to the placebo effects observed across trials weighted by their sample sizes.

**Machine learning classification mood disorders vs. schizophrenia using placebo effect**

For the original sample and for each WBR sample, we used 10-fold cross-validation to evaluate the performance of the model for that sample. The overall performance of the model was estimated over the 1000 bootstrapping samples.

The parameters of the algorithm included class_weight = 'balanced', solver = 'saga', scoring = ‘balanced_accuracy’. For the original data, we used leave-one-out cross-validation (LOOCV) to “predict” whether a testing trial using previously “unseen data” involved schizophrenia (SCZ) or mood disorders collapsed as a single category [comprising: bipolar disorder (BD) depression, BD mania, major depressive disorder MDD]. Within each training, we did an internal 10-fold cross-validation to select the best regularization parameter ‘C’ on a logarithmic scale between 1e-4 and 1e4.

We also conducted multimodal classification using classes of BD depression, BD mania, MDD and SCZ using a similar procedure, with “multi_class” set to ‘multinomial’ and internal cross validation is set to 5-fold. We obtained an averaged balanced accuracy of 0.44 across all models, where the accuracy for BD depression is at 8%, BD mania is at 0%, MDD is at 57% and Schizophrenia is at 83%. The model could not distinguish BD depression, BD mania and MDD, and BD trials were mislabeled as MDD possibility due to sample size imbalance. With respect to the WBR data, it is clear that the model could not distinguish the mood disorders (random classification), but was able to distinguish SCZ from mood disorders.

**Potential neurobiological mechanisms underlying MDD, BD and SCZ**

Placebo effect may involve multiple neurological systems within the brain, such as dopaminergic, serotonergic and opioid systems^1^. Studies on common and distinct neurological signatures of placebo effects in different psychiatric disorders are rare. In MDD, a positron emission tomography (PET) study found that the glucose metabolism was increased in several brain regions in both the placebo group and the group taking fluoxetine, a selective serotonin reuptake inhibitor (SSRI) antidepressant, and no unique regional metabolism was observed in the placebo group^2^. However, both placebo and drug responders showed glucose utilization changes in the nucleus accumbens and the orbito-frontal cortex at one week of treatment, which were associated with anticipation of clinical benefit. The brain region (e.g., nucleus accumbens) related to SSRI response overlaps with the expectation-related dopaminergic circuitry^3^. and led to a speculation that this network might also be involved in other psychiatric disorders, such as SCZ^4^. However, to our best knowledge, there is no study investigating the common and differential neurobiological markers of placebo effect across SCZ, BD and MDD. The current study could pave the way for such studies in the future.

**Further limitations of clinical trials**

Limitations of clinical trials had impact on our results, including potential biases when patients were recruited or informed about the possibility to be assigned to the placebo group or dropped due to unresponsiveness, as well as varied reliability of the outcome measures and the inclusion/exclusion criteria. Our approach provided a preliminary estimation of the placebo effect without quantifying the limitations within each clinical trial, but further thorough quantitative investigation of the clinical trial data will be necessary. Intensive therapeutic contact during clinical trials may lead to additional therapeutic effect compared to regular treatment in the community. This effect may not generalize to usual real-world treatment scenarios. Our study only involved recent trials registered on clinicaltrials.gov, which did not cover historically earlier clinical trials. Earlier trials may focus on specific drugs for a given disorder (e.g., SSRI for MDD) or may have been conducted more in inpatient settings compared to more recent trials. Nevertheless, overall systematic changes of placebo effects in recent trials (e.g., an increase of placebo effect over the years) may not account plausibly for our main finding of differential placebo effects across SCZ, BD and MDD.

**References**

1. Benedetti, F. Placebo effects: From the neurobiological paradigm to translational implications. *Neuron* **84**, 623–637 (2014).

2. Mayberg, H. S. *et al.* The functional neuroanatomy of the placebo effect. *Am. J. Psychiatry* **159**, 728–737 (2002).

3. Benedetti, F. Mechanisms of Placebo and Placebo-Related Effects Across Diseases and Treatments. *Annu. Rev. Pharmacol. Toxicol.* **48**, 33–60 (2008).

4. Holmes, R. D., Tiwari, A. K. & Kennedy, J. L. Mechanisms of the placebo effect in pain and psychiatric disorders. *Pharmacogenomics Journal* vol. 16 491–500 (2016).

**SUPPLEMENTARY FIGURES S1–S3**


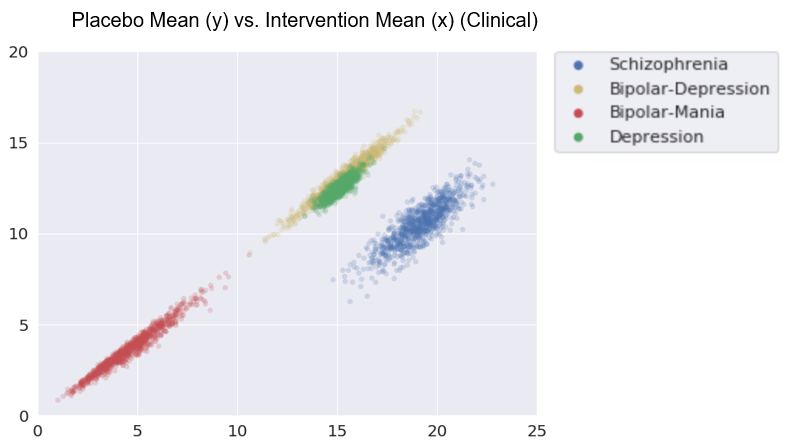


Figure S1. Varied ranges of clinical score changes for schizophrenia, major depression, bipolar depression and bipolar mania made it difficult to compare them directly. The scatter plot is shown with weighted bootstrap resampling for a better demonstration of distributions.


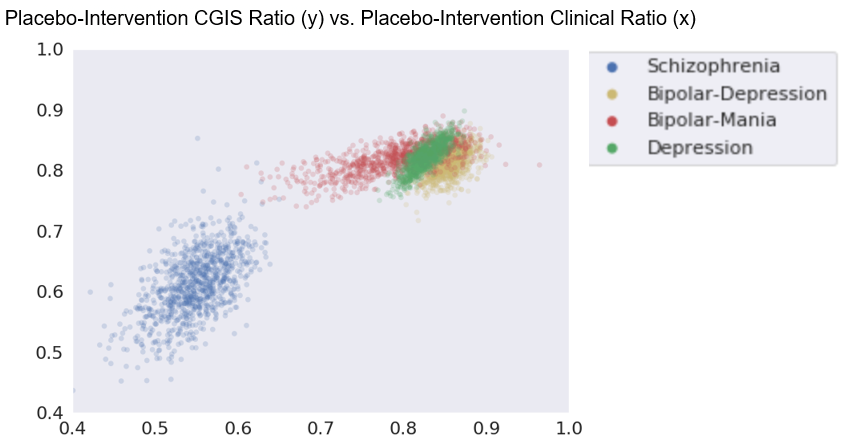


Figure S2. Differential placebo effects for schizophrenia, depression (major depressive disorder), bipolar depression and bipolar mania as measured by the ratio of clinical measurement change for placebo to intervention (x axis) and the ratio of CGI-S change for placebo to intervention (y axis) in a scatter plot. The scatter plot is shown with weighted bootstrap resampling for a better demonstration of distributions.


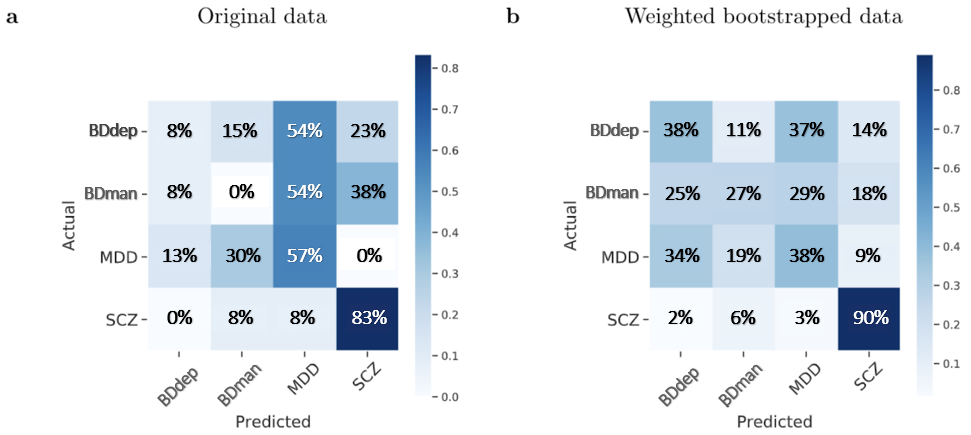


Figure S3. Confusion matrix of L1 penalized multinomial classification model based on a) original data, and b) bootstrapped data. BDdep denotes Bipolar depression, BDman denotes Bipolar mania, MDD denotes Major Depressive Disorder, and SCZ denotes Schizophrenia.

**SUPPLEMENTARY TABLES S1–S6**

**Table S1. Statistics of placebo effect across disorders for bootstrapped samples.**

| **Groups** | **Mean** | **95% CI** | | ***t* (hedge’s g) compared to target group** | | | | | |
| --- | --- | --- | --- | --- | --- | --- | --- | --- | --- |
|  |  | **Lower Bound** | **Upper**  **Bound** | **mean > 0** | **mean < 1** | **MDD** | **BDdep** | **BDman** | **SCZ** |
| **R_clinical_** |  |  |  |  |  |  |  |  |  |
| **MDD** | 0.836 | 0.835 | 0.837 | 1592  (6.5)*** | -312  (-1.3)*** | - | -0.070  (0.00) | -1.9  (0.024) | 141  (1.18)*** |
| **BDdep** | 0.836 | 0.834 | 0.838 | 809  (5.0)*** | -158  (-1.0)*** | - | - | -1.8  (0.03) | 134  (0.96)*** |
| **BDman** | 0.843 | 0.836 | 0.849 | 254  (1.8)*** | -47  (-0.33)*** | - | - | - | 94.8  (0.80)*** |
| **SCZ** | 0.428 | 0.422 | 0.433 | 150  (0.79)*** | -200  (-1.1)*** | - | - | - | - |
|  |  |  |  |  |  |  |  |  |  |
| **R_CGI_** |  |  |  |  |  |  |  |  |  |
| **MDD** | 0.831 | 0.83 | 0.833 | 1250  (5.6)*** | -253  (-1.1)*** | - | 16  (0.12)*** | 7.7  (0.06)*** | 130  (1.1)*** |
| **BDdep** | 0.816 | 0.815 | 0.817 | 1229  (7.9)*** | -277  (-1.8)*** | - | - | -7.7  (0.07)*** | 123  (0.92)*** |
| **BDman** | 0.824 | 0.822 | 0.825 | 1076  (7.6)*** | -230  (-1.6)*** | - | - | - | 124  (0.91)*** |
| **SCZ** | 0.532 | 0.527 | 0.536 | 241  (1.3)*** | -212  (-1.2)*** | - | - | - | - |
|  |  |  |  |  |  |  |  |  |  |
| **R*_CGIbaseline_*** |  |  |  |  |  |  |  |  |  |
| **MDD** | 0.332 | 0.329 | 0.335 | 229  (1.7)*** | -460  (-3.4)*** |  | 29  (0.30)*** | 26  (0.27)*** | 123  (1.3)*** |
| **BDdep** | 0.288 | 0.287 | 0.289 | 570  (4.6)*** | -1407  (-11)*** | - | - | -3.5  (-0.04)*** | -179  (-1.8)*** |
| **BDman** | 0.291 | 0.290 | 0.292 | 488  (4.4)*** | -1190  (-11)*** | - | - | - | -171  (1.8)*** |
| **SCZ** | 0.134 | 0.133 | 0.136 | 193  (1.3)*** | -1247  (-8.7)*** | - | - | - | - |

**Table S2. Results of Bootstrapping Meta-Regression.**

|  | **Scale Ratio** | | | **CGI Ratio** | | | **CGI Baseline Ratio** | | | |
| --- | --- | --- | --- | --- | --- | --- | --- | --- | --- | --- |
|  | **Beta** | **T** | **P** | **Beta** | **T** | **P** | | **Beta** | **T** | **P** |
| **Intercept** | -35.637 | -1.271 | 0.258 | -41.786 | -2.274 | 0.110 | | 0.246 | -0.006 | 0.999 |
| **SCZ MDD** | -0.661 | -5.647 | <0.001 | -0.484 | -5.830 | 0.001 | | -0.271 | -5.319 | 0.043 |
| **BDdepr MDD** | -0.086 | -0.833 | 0.434 | -0.151 | -2.048 | 0.047 | | -0.214 | -4.479 | 0.096 |
| **BDman MDD** | -0.020 | -0.088 | 0.947 | -0.126 | -1.628 | 0.135 | | -0.206 | -4.240 | 0.110 |
| **Industry Academia** | -0.164 | -0.571 | 0.635 | -4.066 | 0.245 | 0.856 | | 0.321 | 0.781 | 0.673 |
| **Phase** | 0.001 | 0.004 | 1.000 | 0.008 | 0.115 | 0.949 | | -0.094 | -2.135 | 0.337 |
| **Residual Symptoms Regular** | -0.321 | -0.961 | 0.460 | -1.725 | -0.538 | 0.591 | | -0.047 | -0.203 | 0.681 |
| **As Co-treatment** | 0.053 | 0.573 | 0.496 | 0.143 | 2.220 | 0.035 | | 0.191 | 4.934 | 0.075 |
| **North America** | 0.331 | 3.755 | 0.001 | 0.126 | 1.961 | 0.103 | | -0.039 | -0.638 | 0.730 |
| **Europe** | 0.046 | 0.536 | 0.542 | -0.056 | -0.929 | 0.541 | | 0.082 | 1.624 | 0.368 |
| **South America** | -0.010 | -0.067 | 0.950 | -0.006 | -0.030 | 0.981 | | 0.054 | 1.401 | 0.403 |
| **Asia** | 0.015 | 0.182 | 0.827 | -0.006 | -0.156 | 0.892 | | -0.036 | -1.122 | 0.508 |
| **Africa** | -0.094 | -0.831 | 0.387 | -0.037 | -0.618 | 0.643 | | 0.027 | 0.637 | 0.736 |
| **Oceania** | -0.005 | -0.082 | 0.916 | 0.009 | 0.103 | 0.919 | | 0.031 | 0.553 | 0.635 |
| **N Intervention** | 0.000 | -0.176 | 0.897 | 0.002 | 2.486 | 0.123 | | 0.000 | -1.096 | 0.552 |
| **N Placebo** | -0.001 | -0.378 | 0.753 | -0.002 | -2.360 | 0.110 | | 0.000 | 0.584 | 0.739 |
| **Age Intervention Mean** | -0.007 | -0.203 | 0.871 | 0.049 | 2.568 | 0.116 | | 0.013 | 1.116 | 0.509 |
| **Age Placebo Mean** | 0.001 | -0.043 | 0.978 | -0.053 | -2.820 | 0.068 | | -0.019 | -1.711 | 0.311 |
| **Sex Ratio Intervention** | -0.106 | -0.788 | 0.642 | 0.030 | 0.219 | 0.897 | | 0.013 | 0.264 | 0.873 |
| **Sex Ratio Placebo** | 0.247 | 2.268 | 0.144 | 0.107 | 1.340 | 0.491 | | -0.016 | -0.256 | 0.885 |
| **Time Duration** | -0.050 | -2.883 | 0.098 | -0.030 | -2.491 | 0.092 | | -0.022 | -3.366 | 0.125 |
| **Start Year** | 0.011 | 0.413 | 0.685 | 0.020 | 0.782 | 0.451 | | 0.002 | 0.012 | 0.993 |
| **Completion Year** | 0.007 | 0.164 | 0.873 | 0.003 | 0.105 | 0.921 | | -0.002 | 0.018 | 0.991 |
| **Number of Facilities** | 0.003 | 2.194 | 0.029 | 0.000 | 0.389 | 0.746 | | -0.002 | -3.214 | 0.146 |
| **Completion Rate** | 0.274 | 1.823 | 0.150 | 0.261 | 2.454 | 0.259 | | -0.109 | -1.090 | 0.530 |
| **Number of Visits** | -0.016 | -1.517 | 0.173 | 0.004 | 0.468 | 0.769 | | 0.019 | 3.242 | 0.172 |
| **Number of Study Arms** | -0.028 | -0.587 | 0.513 | 0.001 | 0.052 | 0.965 | | 0.008 | 0.285 | 0.877 |

**Table S3. Meta-regression of the ratio of clinical measurement change of placebo to intervention.**

|  | **coef** | **standard error** | **t** | **p > \|t\|** | **[0.025** | **0.975]** |
| --- | --- | --- | --- | --- | --- | --- |
| **Intercept** | -10.516 | 28.157 | -0.373 | 0.709 | -66.284 | 45.251 |
| **SCZ MDD** | -0.704 | 0.122 | -5.793 | <0.001 | -0.945 | -0.463 |
| **BDdepr MDD** | -0.108 | 0.103 | -1.053 | 0.295 | -0.311 | 0.095 |
| **BDman MDD** | -0.060 | 0.104 | -0.576 | 0.566 | -0.267 | 0.147 |
| **Industry Academia** | -0.059 | 0.196 | -0.303 | 0.762 | -0.447 | 0.329 |
| **Phase** | 0.060 | 0.089 | 0.675 | 0.501 | -0.116 | 0.236 |
| **Residual Symptoms Regular** | -0.128 | 0.160 | -0.799 | 0.426 | -0.444 | 0.189 |
| **As Co-treatment** | 0.042 | 0.100 | 0.417 | 0.677 | -0.157 | 0.240 |
| **North America** | 0.336 | 0.107 | 3.145 | 0.002 | 0.124 | 0.548 |
| **Europe** | 0.087 | 0.093 | 0.935 | 0.352 | -0.098 | 0.272 |
| **South America** | 0.034 | 0.108 | 0.313 | 0.755 | -0.180 | 0.248 |
| **Asia** | 0.054 | 0.093 | 0.579 | 0.564 | -0.130 | 0.238 |
| **Africa** | -0.063 | 0.121 | -0.520 | 0.604 | -0.302 | 0.177 |
| **Oceania** | -0.096 | 0.170 | -0.567 | 0.572 | -0.433 | 0.240 |
| **N Intervention** | -0.001 | 0.001 | -0.895 | 0.373 | -0.003 | 0.001 |
| **N Placebo** | 0.001 | 0.001 | 0.409 | 0.684 | -0.002 | 0.003 |
| **Age Intervention Mean** | -0.006 | 0.017 | -0.371 | 0.711 | -0.040 | 0.027 |
| **Age Placebo Mean** | 0.002 | 0.017 | 0.131 | 0.896 | -0.032 | 0.036 |
| **Sex Ratio Intervention** | 0.015 | 0.052 | 0.295 | 0.769 | -0.087 | 0.117 |
| **Sex Ratio Placebo** | 0.076 | 0.081 | 0.929 | 0.355 | -0.086 | 0.237 |
| **Time Duration** | -0.041 | 0.017 | -2.395 | 0.018 | -0.075 | -0.007 |
| **Start Year** | 0.006 | 0.028 | 0.216 | 0.829 | -0.050 | 0.062 |
| **Completion Year** | 0.000 | 0.028 | -0.012 | 0.990 | -0.055 | 0.055 |
| **Number of Facilities** | 0.002 | 0.002 | 1.492 | 0.139 | -0.001 | 0.006 |
| **Completion Rate** | 0.047 | 0.093 | 0.504 | 0.616 | -0.137 | 0.230 |
| **Number of Visits** | -0.014 | 0.054 | -0.252 | 0.801 | -0.121 | 0.094 |
| **Number of Study Arms** | -0.014 | 0.012 | -1.161 | 0.248 | -0.037 | 0.010 |

**Table S4. Meta-regression of the ratio of CGI-S change of placebo to CGI-S change of intervention.**

|  | **coef** | **standard error** | **t** | **p > \|t\|** | **[0.025** | **0.975]** |
| --- | --- | --- | --- | --- | --- | --- |
| **Intercept** | -71.976 | 25.161 | -2.861 | 0.005 | -121.994 | -21.958 |
| **SCZ MDD** | -0.556 | 0.101 | -5.524 | <0.001 | -0.757 | -0.356 |
| **BDdepr MDD** | -0.162 | 0.099 | -1.636 | 0.106 | -0.358 | 0.035 |
| **BDman MDD** | -0.146 | 0.100 | -1.458 | 0.148 | -0.345 | 0.053 |
| **Industry Academia** | 0.176 | 0.185 | 0.949 | 0.345 | -0.193 | 0.544 |
| **Phase** | 0.063 | 0.078 | 0.811 | 0.420 | -0.091 | 0.217 |
| **Residual Symptoms Regular** | -0.398 | 0.254 | -1.566 | 0.121 | -0.903 | 0.107 |
| **As Co-treatment** | 0.111 | 0.088 | 1.268 | 0.208 | -0.063 | 0.286 |
| **North America** | 0.195 | 0.082 | 2.370 | 0.020 | 0.031 | 0.358 |
| **Europe** | -0.107 | 0.077 | -1.382 | 0.171 | -0.260 | 0.047 |
| **South America** | -0.036 | 0.077 | -0.468 | 0.641 | -0.188 | 0.116 |
| **Asia** | 0.037 | 0.070 | 0.533 | 0.595 | -0.102 | 0.176 |
| **Africa** | 0.002 | 0.090 | 0.023 | 0.982 | -0.178 | 0.182 |
| **Oceania** | -0.006 | 0.124 | -0.046 | 0.964 | -0.253 | 0.241 |
| **N Intervention** | 0.001 | 0.001 | 1.448 | 0.151 | 0.000 | 0.003 |
| **N Placebo** | -0.002 | 0.001 | -1.714 | 0.090 | -0.004 | 0.000 |
| **Age Intervention Mean** | 0.054 | 0.024 | 2.283 | 0.025 | 0.007 | 0.102 |
| **Age Placebo Mean** | -0.058 | 0.024 | -2.426 | 0.017 | -0.106 | -0.010 |
| **Sex Ratio Intervention** | -0.019 | 0.074 | -0.251 | 0.803 | -0.166 | 0.129 |
| **Sex Ratio Placebo** | 0.174 | 0.084 | 2.078 | 0.041 | 0.008 | 0.340 |
| **Time Duration** | -0.036 | 0.017 | -2.119 | 0.037 | -0.070 | -0.002 |
| **Start Year** | 0.045 | 0.030 | 1.510 | 0.135 | -0.014 | 0.104 |
| **Completion Year** | -0.009 | 0.027 | -0.318 | 0.751 | -0.063 | 0.046 |
| **Number of Facilities** | 0.001 | 0.001 | 0.500 | 0.618 | -0.002 | 0.003 |
| **Completion Rate** | 0.068 | 0.082 | 0.828 | 0.410 | -0.095 | 0.230 |
| **Number of Visits** | 0.002 | 0.042 | 0.036 | 0.972 | -0.082 | 0.085 |
| **Number of Study Arms** | 0.006 | 0.010 | 0.599 | 0.551 | -0.014 | 0.026 |

**Table S5. Meta-regression of the ratio of CGI-S change of placebo to CGI-S baseline of placebo.**

|  | **coef** | **standard error** | **t** | **p > \|t\|** | **[0.025** | **0.975]** |
| --- | --- | --- | --- | --- | --- | --- |
| **Intercept** | -3.358 | 18.941 | -0.177 | 0.86 | -41.894 | 35.179 |
| **SCZ MDD** | -0.279 | 0.060 | -4.620 | <0.001 | -0.402 | -0.156 |
| **BDdepr MDD** | -0.275 | 0.066 | -4.194 | <0.001 | -0.408 | -0.142 |
| **BDman MDD** | -0.265 | 0.066 | -4.033 | <0.001 | -0.399 | -0.131 |
| **Industry Academia** | 0.021 | 0.139 | 0.149 | 0.883 | -0.263 | 0.304 |
| **Phase** | -0.123 | 0.058 | -2.116 | 0.042 | -0.241 | -0.005 |
| **Residual Symptoms Regular** | 0.096 | 0.250 | 0.385 | 0.703 | -0.412 | 0.605 |
| **As Co-treatment** | 0.242 | 0.056 | 4.323 | <0.001 | 0.128 | 0.356 |
| **North America** | -0.022 | 0.065 | -0.342 | 0.734 | -0.155 | 0.110 |
| **Europe** | 0.107 | 0.064 | 1.681 | 0.102 | -0.022 | 0.236 |
| **South America** | 0.076 | 0.055 | 1.374 | 0.179 | -0.036 | 0.188 |
| **Asia** | -0.015 | 0.043 | -0.337 | 0.738 | -0.102 | 0.073 |
| **Africa** | 0.003 | 0.058 | 0.046 | 0.963 | -0.115 | 0.121 |
| **Oceania** | 0.031 | 0.086 | 0.364 | 0.718 | -0.144 | 0.206 |
| **N Intervention** | 0.000 | 0.001 | -0.031 | 0.976 | -0.001 | 0.001 |
| **N Placebo** | 0.000 | 0.001 | 0.310 | 0.758 | -0.001 | 0.002 |
| **Age Intervention Mean** | 0.017 | 0.016 | 1.054 | 0.299 | -0.015 | 0.049 |
| **Age Placebo Mean** | -0.024 | 0.016 | -1.494 | 0.145 | -0.057 | 0.009 |
| **Sex Ratio Intervention** | -0.042 | 0.066 | -0.629 | 0.534 | -0.177 | 0.093 |
| **Sex Ratio Placebo** | 0.030 | 0.061 | 0.491 | 0.627 | -0.093 | 0.153 |
| **Time Duration** | -0.017 | 0.010 | -1.780 | 0.084 | -0.036 | 0.002 |
| **Start Year** | 0.003 | 0.021 | 0.166 | 0.869 | -0.038 | 0.045 |
| **Completion Year** | -0.001 | 0.020 | -0.069 | 0.945 | -0.042 | 0.039 |
| **Number of Facilities** | -0.003 | 0.001 | -3.076 | 0.004 | -0.005 | -0.001 |
| **Completion Rate** | -0.012 | 0.048 | -0.261 | 0.795 | -0.109 | 0.084 |
| **Number of Visits** | 0.014 | 0.029 | 0.466 | 0.644 | -0.045 | 0.072 |
| **Number of Study Arms** | 0.025 | 0.007 | 3.370 | 0.002 | 0.010 | 0.040 |

**Table S6. Raw data used for analysis.**

Due to space constraints. Table S6 is provided in a separate excel file.
